# Democratizing the Development of Chatbots to Improve Public Health: Feasibility Study of COVID-19 Misinformation

**DOI:** 10.2196/43120

**Published:** 2023-12-28

**Authors:** Leigh Powell, Radwa Nour, Randa Sleibi, Hanan Al Suwaidi, Nabil Zary

**Affiliations:** 1 Institute for Excellence in Health Professions Education Mohammed Bin Rashid University of Medicine and Health Sciences Dubai United Arab Emirates; 2 College of Medicine Mohammed Bin Rashid University of Medicine and Health Sciences Dubai United Arab Emirates

**Keywords:** COVID-19, vaccine hesitancy, infodemic, chatbot, motivational interviewing, social media, conversational agent, misinformation, online health information, usability study, vaccine misinformation

## Abstract

**Background:**

Chatbots enable users to have humanlike conversations on various topics and can vary widely in complexity and functionality. An area of research priority in chatbots is democratizing chatbots to all, removing barriers to entry, such as financial ones, to help make chatbots a possibility for the wider global population to improve access to information, help reduce the digital divide between nations, and improve areas of public good (eg, health communication). Chatbots in this space may help create the potential for improved health outcomes, potentially alleviating some of the burdens on health care providers and systems to be the sole voices of outreach to public health.

**Objective:**

This study explored the feasibility of developing a chatbot using approaches that are accessible in low- and middle-resource settings, such as using technology that is low cost, can be developed by nonprogrammers, and can be deployed over social media platforms to reach the broadest-possible audience without the need for a specialized technical team.

**Methods:**

This study is presented in 2 parts. First, we detailed the design and development of a chatbot, VWise, including the resources used and development considerations for the conversational model. Next, we conducted a case study of 33 participants who engaged in a pilot with our chatbot. We explored the following 3 research questions: (1) Is it feasible to develop and implement a chatbot addressing a public health issue with only minimal resources? (2) What is the participants’ experience with using the chatbot? (3) What kinds of measures of engagement are observed from using the chatbot?

**Results:**

A high level of engagement with the chatbot was demonstrated by the large number of participants who stayed with the conversation to its natural end (n=17, 52%), requested to see the free online resource, selected to view all information about a given concern, and returned to have a dialogue about a second concern (n=12, 36%).

**Conclusions:**

This study explored the feasibility of and the design and development considerations for a chatbot, VWise. Our early findings from this initial pilot suggest that developing a functioning and low-cost chatbot is feasible, even in low-resource environments. Our results show that low-resource environments can enter the health communication chatbot space using readily available human and technical resources. However, despite these early indicators, many limitations exist in this study and further work with a larger sample size and greater diversity of participants is needed. This study represents early work on a chatbot in its virtual infancy. We hope this study will help provide those who feel chatbot access may be out of reach with a useful guide to enter this space, enabling more democratized access to chatbots for all.

## Introduction

### Background

Chatbots are becoming more commonplace in our daily lives, especially in fields such as consumer marketing, customer support, education, and health care, and have significantly increased in recent years [1,2]. Chatbots enable users to have humanlike conversations on various topics. They can vary widely in complexity and functionality, ranging from simple information-giving chatbots to those using artificial intelligence to understand human language input [3]. There is a great diversity of chatbots available today, ranging in abilities, features, and complexity. A hierarchy of chatbots has been proposed [4,5], which classifies chatbots according to their ability to use algorithms or artificial intelligence or both to recognize the context of language as it is written in a real-time conversation, called natural language processing (NLP), and respond with greater specificity. Chatbots higher on the classification scale have more advanced NLP, promoting a greater likelihood of mimicking an actual conversation. The more advanced the chatbot, the greater the need for specialized technical expertise to build and maintain it.

Chatbot development platforms have come a long way, enabling those without technical expertise to use visually interactive approaches to develop chatbots and provide a simplified deployment approach that allows easy integration into social media platforms [6]. As technology advances, the opportunity for low- and middle-resource environments to move into the chatbot space increases. In their study describing emerging research needs in chatbots, Følstad et al [7] stated that a priority area is democratizing chatbots to all. Democratizing chatbots means removing barriers to entry, such as financial resources, technical resources, or specialized human resources, to help make chatbots a possibility for the wider global population. The goal of democratizing chatbots is to improve access to information, help reduce the digital divide between nations, and improve areas of public good [6,7].

An area that has seen expanded activity in recent years is chatbots for health care and health communication. Chatbots are increasingly used in health care to address various concerns, from simple to complex. Chatbots in the health space are typically domain specific, deployed for a particular area of focus [2]. Common uses for chatbots include simple tasks, such as providing tracking and reminders to support medication and appointment adherence [8,9]. However, more advanced chatbots are implemented to support and promote more complex health concerns, such as mental health support [10-12], smoking cessation [13], and promoting physical health and nutrition [9].

Chatbots that address these more complex health concerns often integrate a behavior change model into the conversation. This ensures that the chatbot does not simply deliver information, the least efficient way to impact health behaviors [14], but converses with the participant to maximize the opportunity for behavior change. These chatbots are often more advanced in functionality, such as NLP. The more advanced the chatbot, the greater the need for specialized technical and human resources, creating additional costs, which restrict chatbots to only affluent nations that can fund such projects. However, with the advent of simplified and low-cost development platforms enabling even nonprogrammers to build chatbots, there is an opportunity to democratize chatbots to all nations, including those with low resources. In these nations, health communication chatbots may make the most impact, potentially alleviating some of the burden on health care providers and health care systems to be the sole voices of outreach to public health. For example, studies have reported building a chatbot that asks diagnosis questions to help rule out or detect possible COVID-19 cases, thereby reducing the number of patients coming into primary care [15], or chatbots that can diagnose a disease and provide some information about it before consulting a physician, thereby reducing health care costs and providing medical information from a credible source [16,17]. Other chatbots aim to promote healthy lifestyles and public health education by delivering nutritional education [18] and continued care at home for geriatric patients after hospital discharge [19]. All these chatbots and more serve as virtual assistants ensuring patient care and education without burdening health care systems.

Our domain of focus is COVID-19 vaccine misinformation. The public’s ability to receive information, communicate their needs, connect with others, and mobilize community engagement are all factors that can impact the success of health communication initiatives [20]. The COVID-19 pandemic is an example of a worldwide impact in which social media was used to propagate misinformation regarding the virus and the vaccine [21]. Inaccurate and false information severely impacts public health, delaying individual health choices to take preventative measures, such as mask wearing or social distancing, and having broader impacts on vaccine uptake [22]. Even considering multiple personal and business accounts, a significant number of the population use and interact on social media [23]. Social media users increasingly use these platforms as information sources, rapidly consuming and sharing information [24]. Misinformation about COVID-19 has been prevalent on social media since the start of the pandemic [25], negatively impacting public trust in new COVID-19 vaccines and delaying, and even denying, uptake in various communities [22].

### Objectives

This study explores the feasibility of developing a chatbot using approaches that are accessible in low- and middle-resource settings. These approaches include using technology that is low cost, can be developed by nonprogrammers, and can be deployed over social media platforms to reach the broadest-possible audience. In addition, the technology used does not require a specialized technical team, uses freely available and accurate knowledge bases, and is developed using evidence-based practices to create a conversational model that integrates the potential for a change in health behaviors.

The paper answers the following research questions:

- Is developing and implementing a chatbot addressing a public health issue with only minimal resources feasible?
- What is the participants’ experience with using the chatbot?
- What kinds of measures of engagement are observed from using the chatbot?

## Methods

### Ethical Considerations

The Mohammed Bin Rashid University (MBRU) Institutional Review Board (IRB) approved this study (approval no. MBRU IRB-2021-67). Consent was obtained from the participants.

### Study Design

Low-resource environments face a myriad of challenges that can prevent them from entering the chatbot space. These include lacking available human, technical, or specialized resources. Another barrier of entry is the lack of exposed details about how to design a chatbot, an area not often elaborated on in published studies. This section reports on the considerations for the technical environment, the project team, the process of conversation design, and implementation on the technology platform.

### Choosing a Platform

We carefully reviewed several technology platforms using the following requirements: (1) low recurring monetary costs, (2) a simplified development interface that could be easily used by an individual with little to no technical expertise, and (3) cloud hosting to enable easy deployment and avoid the need for specialized hardware. As a result, we selected ManyChat [26], a cloud-based platform with an easy-to-use interface, simple and direct integration into social media platforms, and low and predictable recurring costs.

**Figure 1:**
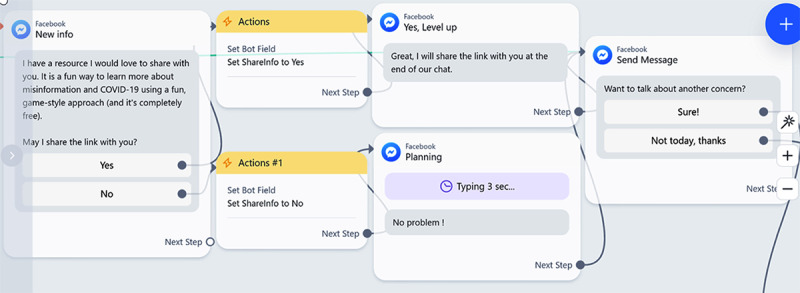
Example of conversational connections built using the ManyChat user interface.

ManyChat simplifies conversation development using interactive visual displays of conversational decision trees, enabling users to drag actions and responses (see Figure 1). ManyChat also seamlessly integrates into major social media platforms (ie, Instagram, Telegram, WhatsApp, Facebook Messenger). We selected Facebook Messenger as our deployment platform because it is freely available and widely used worldwide and Google Sheets as a means to collect data from each conversation.

### Conversation Design Process

The core project team for this study consisted of 2 educational experts and 1 research assistant, with consultation advice from 2 health professionals with subject matter expertise in vaccinations and 1 researcher with a focus on computer science.

#### Behavior Change Model

The purpose of any health communication initiative is to change behavior; the aim is the same when using the medium of a chatbot. Integrating a behavior change model into conversation design is an emerging trend in chatbots designed to promote health communication [27-30]. Although behavior change was outside this study’s scope, we wanted to ensure that the foundations of behavior change were integrated into our conversation model. Many models have been established and tested for patient education and behavioral change. Motivational interviewing (MI) is a behavioral change model used by health care professionals and has been found to be effective. MI has also been used as a foundation in chatbots [10,13]. The MI process includes asking questions to elicit participants’ statements about their beliefs. Conversations in MI are examined by looking at the participants’ statements and identifying them as being indicative of “change talk” or “resistance” [14,31]. MI has a 4-phase approach: engaging, focusing, evoking, and planning [14]. Engaging aims at building a rapport with the participant. Focusing allows the determination of the problem or the identification of the concern. Evoking is when change talk is investigated, and planning reinforces commitment and actions.

#### Chatbot and Participant Persona Development

We collectively developed personas for both the chatbot and potential participants [32,33].

#### Chatbot

**Figure 2:**
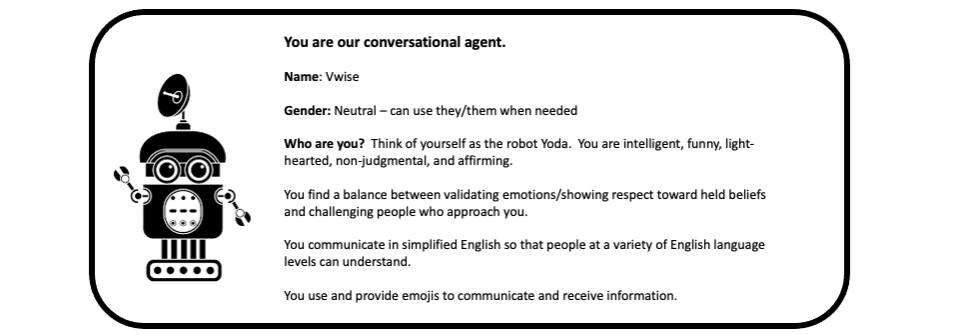
Persona for VWise.

Characteristics of the chatbot persona included name, gender, personality, and communication style (Figure 2). We selected to use a robot persona as a physical representation, owing to the multicultural environment in the United Arab Emirates, in which many different cultures and styles of dress are seen based on nationality or religious affiliation. Therefore, we named our chatbot VWise. Following VWise’s persona, we developed a list of affirmations, demographic details, and jokes to implement during the conversation.

#### Participants

**Figure 3:**
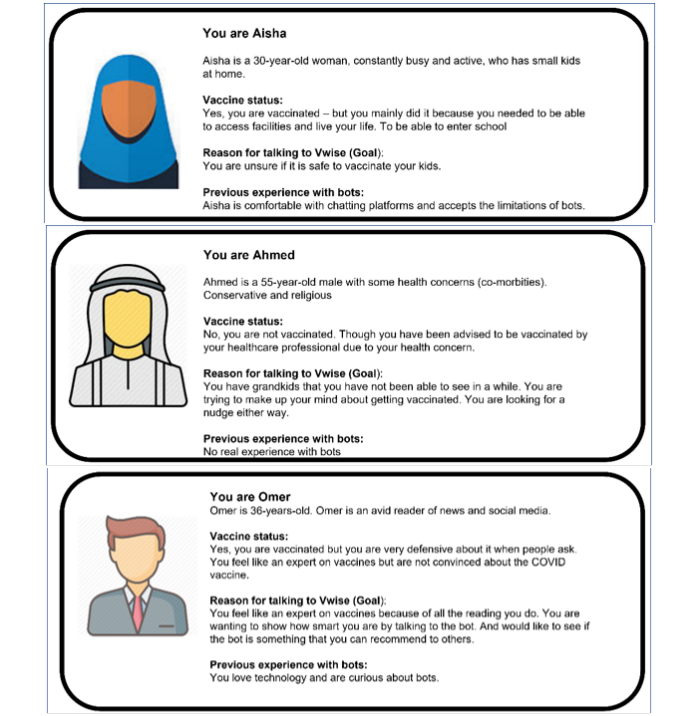
Examples of user personas guiding our conversation development.

We also developed and used participant personas to guide our early dialogues. As personas are meant to be grounded in real data, we determined characteristics collaboratively using real-life examples of people we had encountered. These characteristics included variations in vaccination status, perspectives about vaccination, vaccine knowledge, sources of information, gender, age, and comfort with technology (Figure 3) [33].

#### Sample Dialogues

Using our participant and chatbot personas, we conducted mock conversations, where one team member was assigned the role of a persona and another member played the role of the chatbot. These early conversations were used as a test bed to help them understand the flow of a conversation and help us develop chatbot utterances, affirmations, and refine VWise’s personality. The remaining team members took notes, and all conversations were recorded and transcribed. Although behavior change was beyond the scope of this study, promoting behavior change was the ultimate goal, and so our conversation model was built with this in mind. MI is rooted in empathy toward patients. Therefore, it was important for us to design a conversation sequence that ensured conversations affirmed what the participants said, and expressed empathy before asking evoking questions or presenting new information.

Several iterations of sample dialogues were created, and mock conversations were held with volunteer colleagues outside the research team, which were also recorded, transcribed, and coded similarly.

#### Conversation Tracks and Personalization

Based on the variety of COVID-19 vaccination concerns that arose during mock conversations, we decided to limit the conversation to mRNA vaccines. Creating a knowledge base for a chatbot can be a resource-intensive endeavor [34,35]. As such, we used freely available frequently asked questions (FAQs) from the World Health Organization (WHO) [36] and the Centers for Disease Control and Prevention (CDC) [37] for our knowledge base. These FAQs were rewritten into a conversational style during the design process, and conversation tracks were developed based on each concern. The rewritten information was then reviewed by 2 health professionals with expertise in vaccinations to ensure accuracy of the information.

We addressed 5 concerns related to mRNA COVID-19 vaccines through VWise in this pilot:

- mRNA vaccines were developed and approved too quickly.
- Are mRNA vaccines safe?
- What is mRNA?
- mRNA might change my DNA.
- mRNA might cause fertility issues.

A conversation track was built for each of these concerns, and each was put into a sample dialogue and trialed with volunteer colleagues. Colleagues included native and nonnative English speakers, which enabled us to refine our language, phrasing, and the chatbot’s personality to help make VWise accessible to the broadest-possible audience. Using sample dialogues also aided in refining the flow of the conversation to help make it more personal and engaging to the participant. Personalizing elements of chatbot conversations promote engagement in chatbots used in health education and commercially [38,39].

**Figure 4:**
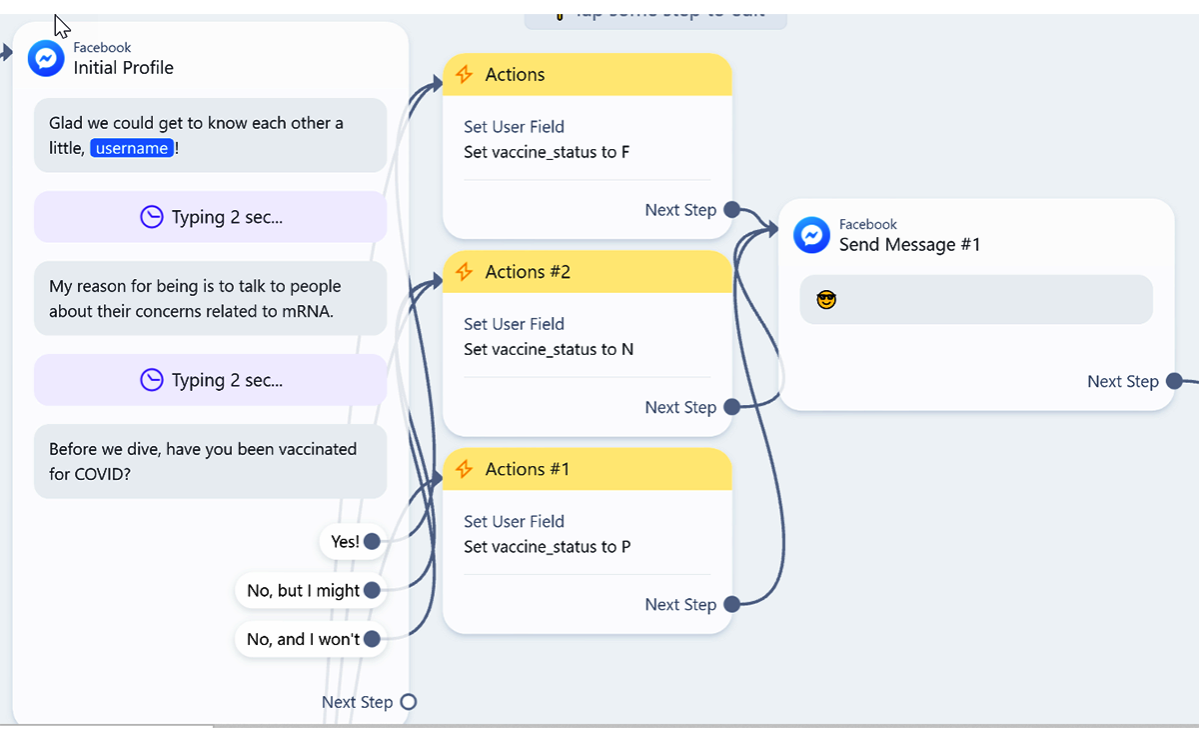
Conversation flow storing the vaccination status for later conversation tracks.

**Figure 5:**
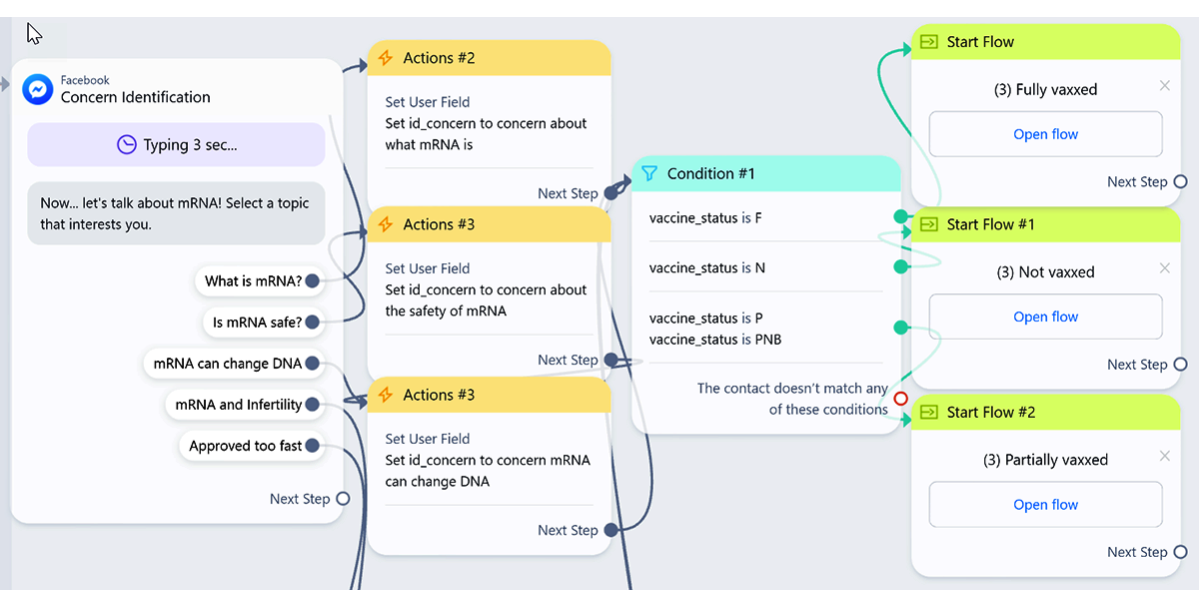
Conversation flow storing participant concerns and launching conversation tracks based on the vaccination status.

**Figure 6:**
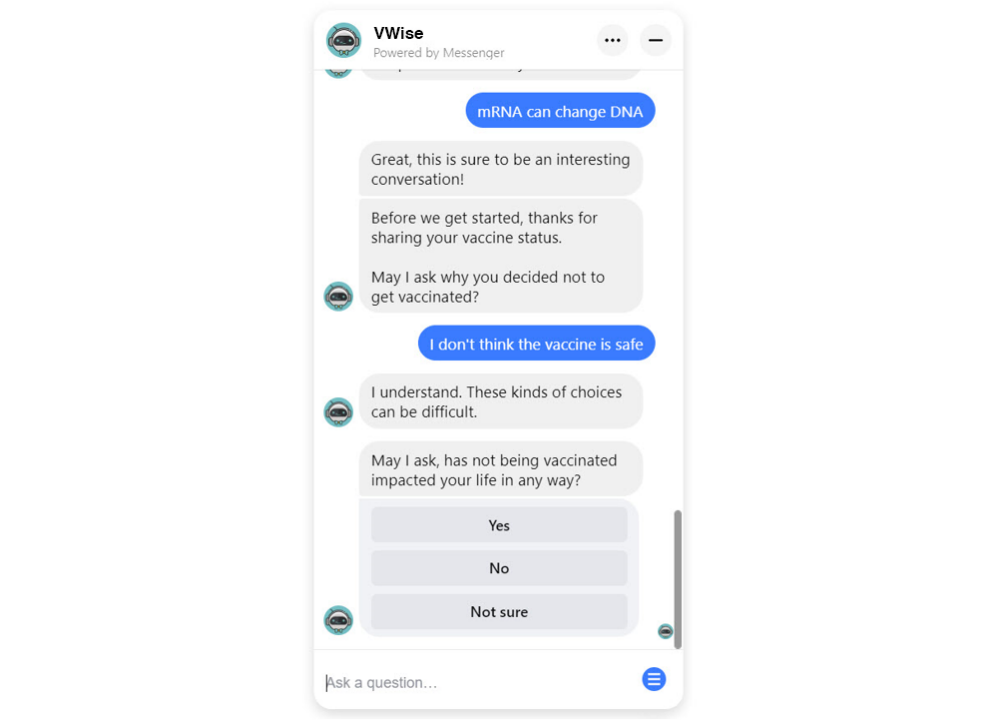
Screenshot of conversation personalization in VWise for a participant who is not vaccinated.

NLP is not yet feasible for those without access to specialized technical resources. So, our conversation was designed to leverage variables and branching to achieve some personalization. Elements that were personalized included vaccination status (ie, conversation tracks based on fully vaccinated, partially vaccinated, unvaccinated status) as well as answers to demographic questions to build rapport (Figures 4-6).

As our conversation tracks developed, we sought to balance the user and chatbot interactions to ensure a free-flowing exchange during the conversation. As such, we chose to start the conversation with an information-seeking question, a process referred to as a “call to action” [40]. Further refining of the dialogues included aspects of the user interface, using buttons and multiple-choice options to help account for the lack of NLP. In addition to phrasing questions in ways that enabled participants to express their concerns, fears, and misconceptions, which is a fundamental aspect of MI [41], we also delivered affirmations and responses that could be somewhat universal to anything said by the participants [42].

### Conversation Design Implemention

**Figure 7:**
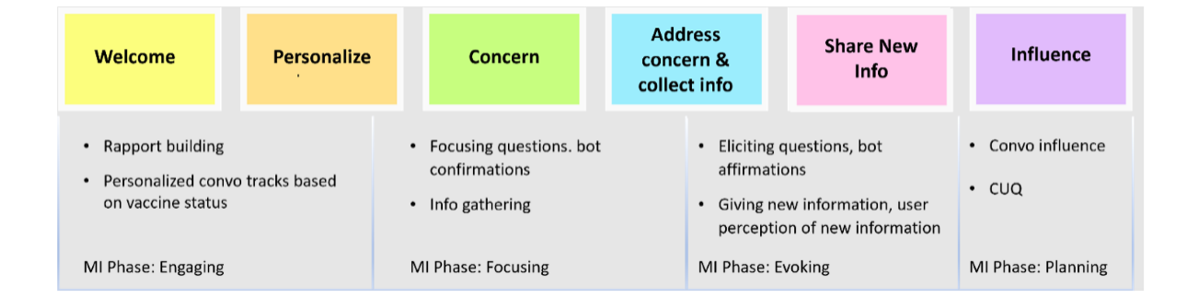
Our conversation design implemented in VWise. CUQ: Chatbot Usability Questionnaire; MI: motivational interviewing.

**Figure 8:**
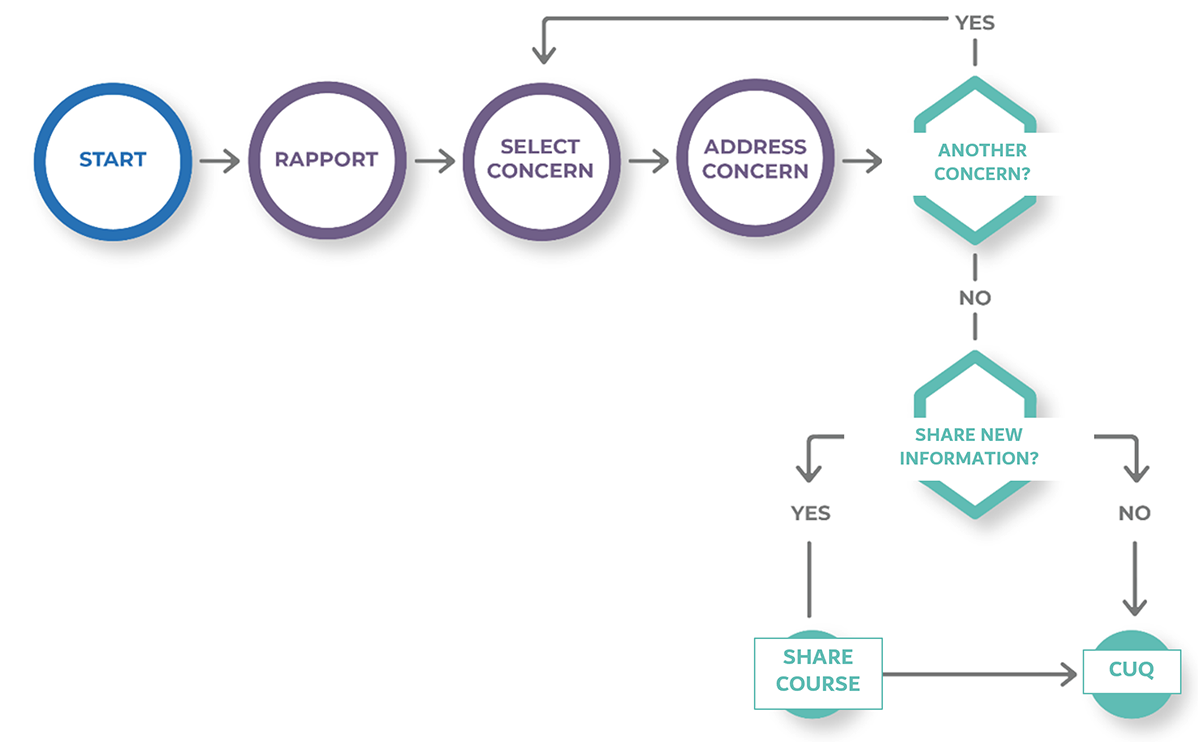
Conversation flow in VWise. CUQ: Chatbot Usability Questionnaire.

A conversation implemented in VWise consists of multiple-choice logic trees with preplanned answers and conversational branches that allow for some personalization of the conversation without the need for NLP. Figure 7 represents the different stages of a conversation. The first 2 phases (welcome and personalize) use rapport building to learn demographic details about the participant, including vaccination status, and share details about VWise as a character. The participant then identifies the area of concern to be addressed using buttons to represent each concern. Next, VWise addresses the concern by exchanging information, which includes gathering additional details about the participant, such as their perceptions about vaccination and the methods of how they typically receive information. Once the concern is addressed, VWise offers an opportunity to address another concern (Figure 8), and the conversation loops back until the participant responds “no.” VWise then asks whether to share further information in the form of a free online course, for which the participant can select “yes” or “no.” Finally, the conversation ends with understanding the participant’s perception of the chatbot and the influence of the conversation itself. Data from the conversation are stored and then written to a Google Sheet at the end of each stage.

Participant perception was investigated using the Chatbot Usability Questionnaire (CUQ) [43], a validated tool that assesses different aspects of usability: chatbot’s personality, onboarding, user experience, and error handling. The questionnaire consists of 16 questions, 8 (50%) related to the positive aspects and 8 (50%) to the negative aspects of chatbot usability. The CUQ was embedded in the chat, and scores were calculated using a Microsoft Excel spreadsheet provided by the questionnaire developers. The CUQ score is calculated out of 100. The CUQ developers have designed it such that the scores are comparable to the System Usability Scale (SUS) [44], where scores >68 are considered above average. The process data collected are further described in the Case Study section.

Next, we present a case study detailing the pilot for VWise.

### Case Study

#### Study Design

This study used a convenience sampling approach in which invitations were sent to colleagues at the institution and to those in the research team’s networks. The final number of participants was 33. Participants were provided with an explanation about the aim of the study, the nature of participation, and a description of how to use the chatbot. A consent form was embedded in the conversation, and only participants who selected “yes” were allowed to proceed with the chat. Those who selected “no” were provided with a link to a free educational resource. Inclusion criteria were adults with English language proficiency, digital literacy, and the ability to provide consent.

#### Data Collection and Analysis

Since ManyChat does not store conversations in their entirety, relevant participant responses were first stored in variables and then mapped to a preconfigured Google Sheet that became our data set for analysis. No identifying data, Facebook profile data, or any background data generated by ManyChat were included in the data set. Two independent researchers deductively coded participant responses to qualitative questions. All other data were quantitatively analyzed.

## Results

Our results are reported in 2 sections, (1) user engagement and (2) user experience.

### User Engagement

This section describes how participants engaged with VWise during the pilot, including their journey through the conversation, concerns selected, and points of attrition.

#### Participant Journey

**Figure 9:**
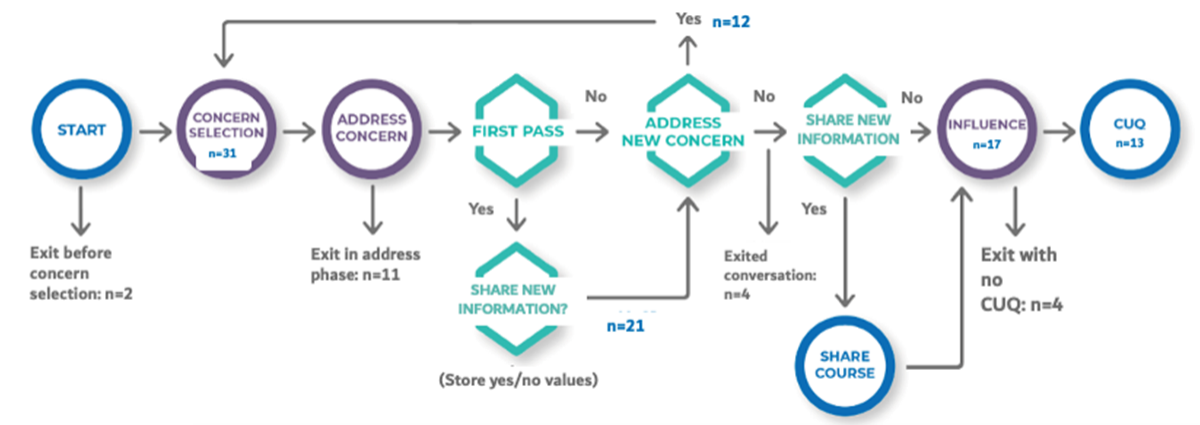
Participants’ journey through VWise in the pilot, with attrition rates. CUQ: Chatbot Usability Questionnaire.

Here is a narrative representation of Figure 9, which represents the journey of our pilot participants through VWise, including the areas of attrition at various points in the conversation.

In total, 33 participants began a conversation with VWise, but 2 (6%) exited the conversation prior to the “concern selection” phase, making our sample size 31 (94%). Of these 31 participants, 10 (32%) exited the conversation during the initial “addressing concern” phase. VWise is designed to address 1 concern at a time, allowing participants to loop back and select another concern. The remaining participants (n=21, 68%) were asked whether they would be interested in receiving a free information source about misinformation. Of these 21 participants, 12 (57%) decided to address another concern. No participants elected to address a third concern.

Furthermore, 8 (38%) of the 21 participants exited the conversation before the end. All participants chose to address only 1 concern (4, 50%, exited after addressing the concern and 4 (50%) without completing the CUQ). In addition, all 12 participants who selected a second concern completed the conversation up to the “influence” phase, with 9 (75%) reaching the end of the conversation by completing the CUQ.

In the end, 17 (55%) of 31 participants reached the “influence” phase, with 13 (76%) completing the entire conversation by filling out the CUQ.

#### Vaccination Status of Participants

Overall, we had a higher number of fully vaccinated participants. Of the 31 participants, 18 (58%) were fully vaccinated, 9 (29%) were partially vaccinated, and 4 (13%) were unvaccinated.

Of the 17 (55%) participants who reached the “influence” phase of the conversation, 11 (65%) were fully vaccinated, 5 (29%) were partially vaccinated, and 1 (6%) was unvaccinated.

#### Concerns

Participants were provided with a list of 5 concerns to choose from in the form of clickable buttons. Table 1 presents the distribution of concerns among the participants.

**Table 1:** Distribution of 5 concerns among the participants (N=33).

Concern	Participants, n (%)
mRNA vaccines were developed and approved too quickly.	5 (15)
Are mRNA vaccines safe?	3 (9)
What is mRNA?	14 (42)
mRNA might change my DNA.	5 (15)
mRNA might cause fertility issues.	4 (12)

Some of the concerns VWise addressed were quite complex, containing many pieces of relevant information. For these concerns, we divided the large and complex responses in the WHO and CDC data into smaller, more engaging chunks of information. VWise asked the participants whether they would like to hear more information between each chunk of information. We interpreted each affirmative response to provide more information as a measure of engagement. In our analysis, all the participants who selected the concerns “mRNA vaccines were developed and approved too quickly” and “mRNA might cause infertility” opted to receive all 5 chunks of information about the concern.

#### Qualitative Indicators of MI

##### Change Talk vs Resistance

VWise asked open-ended questions to hear participants’ perceptions and concerns in their own words, a technique used in MI known as using evoking questions [45]. Evoking questions in MI are used to understand a participant’s willingness to change behavior. Although behavior change was outside the scope of this study, we wanted to understand whether our conversation elicited any evidence of “change talk” or “resistance.” Indications of resistance in MI include arguing, interrupting, negating, or ignoring [14]. Responses were manually and deductively coded qualitatively by 2 independent researchers, who then discussed their codes to reach consensus.

The conversation flow provided partially vaccinated and unvaccinated participants with 2 opportunities to elicit a willingness to change:

- “Has not being vaccinated/not getting your booster shot impacted your life in any way?” Partially vaccinated (9/12, 75%) and unvaccinated (3/12, 25%) participants were asked about the impact of not being vaccinated/fully vaccinated on their life. Options were “yes,” “no,” and “not sure.” VWise asks users who respond with “yes” a follow-up, open-ended question: “In what ways has your life been impacted?” However, all 12 (100%) participants responded with a resistance answer (ie, “no,” “not sure”), so no participant was asked the qualitative follow-up question about how their life was impacted.
- “If you were to close your eyes and think about your daily life and routine, in what ways might your life be different if you were to get fully vaccinated?” All 12 (100%) partially vaccinated and unvaccinated participants were asked this question, and responses were coded as either “change talk” or “resistance”. Only 2 (17%) participants expressed any form of change talk, and both were unvaccinated (Table 2).

**Table 2:** Responses of partially vaccinated and unvaccinated participants responses.

Vaccination status	Response	Code
N^a^	“I could see my friends and play hockey”	C^b^
N	“Cannot think of a way”	R^c^
N	“Less PCRs^d^”	C
P^e^	“Not sure”	R
P	“Forgetting things more?”	R
P	“No”	R
P	“Don’t know”	R
P	“No”	R
P	“My [life] will be normal as already take[n] 2 doses, that’s why I don’t think that the booster [will] make any difference”	R
P	“I cannot find any difference if still I will be in [the] ICU^f^ even with 2 doses”	R
P	“What do you think”	R
P	“Lol”	R

^a^N: not vaccinated.

^b^C: change talk.

^c^R: resistance.

^d^PCR: polymerase chain reaction.

^e^P: partially vaccinated.

^f^ICU: intensive care unit.

##### Influence of Conversations

The planning phase of MI typically involves using the participants’ language to turn their words into action. Given the low-tech nature of the chatbot (ie, no NLP), we asked an open-ended question to try and understand whether any participant might have changed their perception of their concern or getting an mRNA vaccine: “Before you go, I would really like to know how this conversation has influenced your opinion about mRNA. What would you like to share with me? Responses were coded and categorized as positive, negative, or neutral (Table 3). Only 17 (52%) participants made it to this stage in the conversation, with 10 (59%; n=6, 60%, fully vaccinated and n=4, 40%, partially vaccinated) expressing a positive opinion, 1 (4%; partially vaccinated) expressing a neutral opinion, and 6 (37%; n=5, 83%, fully vaccinated and n=1, 17%, unvaccinated) not answering.

**Table 3:** Coded responses to the question “Before you go, I would really like to know how this conversation has influenced your opinion about mRNA. What would you like to share with me?” (N=17).

Influence of conversation	Fully vaccinated (n=11), n (%)	Not vaccinated (n=1), n (%)	Partially vaccinated (n=5), n (%)
No response	5 (45)	1 (100)	0
Neutral	0	0	1 (20)
Positive	6 (55)	0	4 (80)
Negative	0	0	0

#### User Experience

##### Participant Demographics

Participants were asked their name, age, and location, with names being stored and used to personalize welcome responses. The mean age self-reported by participants (n=28, 85%; n=5, 15%, participants did not respond or provided unreal answers, eg, 99 years) was 36.6 years (SD 10.02). Table 4 presents the self-reported location details of participants.

**Table 4:** Self-reported location of participants (N=33).

Location	Participants, n (%)
Afghanistan	1 (3)
Bermudas	1 (3)
Canada	2 (6)
India	1 (3)
Morocco	4 (12)
Nepal	1 (3)
Switzerland	1 (3)
Tunis	1 (3)
United Arab Emirates	19 (58)
No response	2 (6)

Fully vaccinated participants accounted for around 58% (n=19) of the sample, 12% (n=4) were not vaccinated, and 30% (n=10) were partially vaccinated. Vaccinated (fully and partially) participants (n=29, 88%) were asked to rate the importance of getting a vaccine on a scale of 0-10. The mean score was 7.5 (SD 2.8), with 72% (n=21) of the 29 participants rating the importance to vaccinate as ≥6 (Table 5). Fully vaccinated participants (n=19, 58%) rated the importance of getting a vaccine as ≤5 (n=4, 21%) and ≥6 (n=15, 79%). Partially vaccinated participants (n=10, 30%) rated the importance of getting a vaccine as ≤5 (n=4, 40%) and ≥6 (n=6, 60%). Unvaccinated participants were not asked this question.

**Table 5:** Distribution of participant responses to the question “On a scale of 0-10, how important to you was it to get vaccinated (0=not important at all, 10=very important)?” (N=29).

Score for perceived importance of getting vaccinated (scale 0-10)	Fully vaccinated (n=19), n (%)	Partially vaccinated (n=10), n (%)
≤5	4 (21)	4 (40)
≥6	15 (79)	6 (60)

#### The Chatbot Usability Questionnaire

The CUQ was included at the end of the conversation. Of the 17 (52%) participants who concluded the chat, 13 (76%) filled out the CUQ. The mean score was 70.9 (SD 19.4), and the median score was 78.1, with the lowest and highest scores being 34.4 and 95.3, respectively. The mean score was higher than the standard mean SUS score of 68. Five positive aspects of the chatbot’s personality scored ≥4 on a 5-point Likert scale: (1) realistic and engaging personality (mean score 4.1, SD 1.2), (2) welcoming during initial setup (mean score 4.4, SD 0.8), (3) explained its scope and purpose well (mean score 4.3, SD 0.6), (4) was easy to navigate (mean score 4.0, SD 1.2), (5) and was easy to use (mean score 4.3, SD 0.9). The remaining 3 positive aspects scored as follows: (1) understood me well (mean score 3.4, SD 1.3); (2) responses were useful, appropriate, and informative (mean score 3.8, SD 1.1); and (3) coped well with any errors or mistakes (mean score 3.0, SD 1.1). All negative aspects of the chatbot scored <3 (Table 6).

**Table 6:** Mean CUQ^a^ score of each aspect.

Question	Score, mean(SD)
The chatbot’s personality was realistic and engaging.	4.1 (1.2)
The chatbot seemed too robotic.	2.8 (1.5)
The chatbot was welcoming during initial setup.	4.4 (0.8)
The chatbot seemed unfriendly.	1.5 (0.8)
The chatbot explained its scope and purpose well.	4.3 (0.6)
The chatbot gave no indication as to its purpose.	2.1 (1.1)
The chatbot was easy to navigate.	4.0 (1.2)
It would be easy to get confused when using the chatbot.	2.1 (1.1)
The chatbot understood me well.	3.4 (1.3)
The chatbot failed to recognize a lot of my inputs.	2.9 (1.5)
Chatbot responses were useful, appropriate, and informative.	3.8 (1.1)
Chatbot responses were irrelevant.	2.4 (1.2)
The chatbot coped well with any errors or mistakes.	3.0 (1.1)
The chatbot seemed unable to handle any errors.	2.5 (1.2)
The chatbot was easy to use.	4.3 (0.9)
The chatbot was complex.	1.6 (0.8)

^a^CUQ: Chatbot Usability Questionnaire.

In addition to the CUQ, some participants voluntarily sent us their feedback via email. We noted a diversity in the feedback regarding the personality of the chatbot.

Some of the positive feedback included:

Bot is friendly

VWise is quick to respond.

Engages with the participant

I like the conversational use of language.

Clever chatbot with precise answers!

Not all comments about VWise were positive:

A bit too friendly

VWise seems a funny character. Please revisit. [H]ere are areas you can avoid having some funny comments and emojis. Not everyone like too much fun when discussing serious/important information.

I felt like the intro was too long and a little “extra friendly”. It could be shortened, but the extra friendly could be great if you’re targeting kids and younger adults when sharing information about the vaccine.

We also received feedback about the content of the conversation from participants. Early positive feedback included:

It’s good to have informed by this topic so now I have [an] idea of what mean mRNA.

More confident to take the vaccine

Criticisms included a desire to be pointed to additional resources:

When sharing the answer about the relation between taking the vaccine and infertility, it might’ve been better to share a source perhaps [than] just saying “studies show no connection between them”.

In addition, participants highlighted the need to create a conversation flow that focuses more on a 2-way exchange of interactions:

When I used it, it felt more the bot wants to know about myself as opposite to I want to use the bot to know more about something.

## Discussion

### Principal Findings

This study sought to address the possibility of democratizing chatbot access to all by exploring the feasibility of developing a chatbot for public health communication using readily available resources and technology that would be accessible in low- and middle-resource settings. We explored this through a detailed description of the design and development considerations for our chatbot and by presenting a case study describing our initial pilot with 33 people who engaged in a conversation about COVID-19 vaccine misinformation.

Our study highlights that even in low-resource environments, the ability to develop a functioning and low-cost chatbot is feasible. A high level of engagement with the chatbot was demonstrated by the large number of participants who stayed with the conversation to its natural end and requested to see the free online resource, who selected to view all information about a given concern, and who returned to have a dialogue about a second concern. More than half of the participants (52%) continued the conversation till the end, and around 36% went for a second chat. These numbers are promising and suggest that with further improvement, the retention rate could be high.

Participants’ responses to the CUQ were positive. Emailed comments from participants revealed a need to work on the conversational flow as well as the chatbot’s personality (ie, extra friendly, a funny character). The discrepancy in the reception to the chatbot’s personality could be attributed to the cultural diversity of the participants. Nißen et al [46] recently noted that demographic differences, especially age, may be a determiner of receptivity to a chatbot, helping determine whether a bond between participant and chatbot might be formed. An emerging area of exploration is the development of personality-adaptive chatbots, in which the chatbot’s personality is tailored to an individual user based on key characteristics [47]. Future work on VWise could investigate whether the ability to create personality-adaptive chatbots is conducive to environments in which specialized resources are scarce.

Although behavior change was outside the scope of this study, we observed participants’ responses that could be construed as willingness or resistance to change. These observations could not be processed in real time (ie, only through post hoc manual coding), so we believe that the results, at the minimum, demonstrate that our conversation flow is promising and stimulates thought processes. This could be a precursor to behavior change. Future iterations should seek to take advantage of the advanced features of ManyChat, such as keyword detection, designed to help simulate an NLP experience. Another avenue for exploration is to use a hybrid approach, in which non-NLP chatbots serve to conduct an initial consultant, helping identify candidates for further intervention. For example, Lee et al [48] followed a hybrid approach in which a low-tech chatbot was used as a mediator for patients to self-disclose mental health needs before approaching a mental health professional. In our case study, a wealth of information about participants was collected, making branching scenarios possible, in which specific participants are pushed toward professionals or educational interventions.

Finally, to help further the aim of democratizing chatbot access for all, we recommend that future studies expose and labor the design and development processes and technology choices for their chatbots to enable others to reproduce their work. When consulting the literature to guide this project, only a few studies elaborated on the process of designing a conversational model or included recommendations for a smooth user experience [10,49,50]. Industry is further ahead in this area and was used more as a guide for this study [33,51-53]. Research should seek to catch up to industry by sharing the best practices and processes through published, peer-reviewed work.

### Limitations

This early study explored the feasibility of an approach to developing chatbots in low-resource environments. However, this study has many limitations.

This pilot was not a controlled study. Convenience sampling was used, and our sample size was quite small, consisting primarily of fully vaccinated people and with fewer respondents to the CUQ. Therefore, due to the limited scope of this paper (ie, feasibility), our results are largely descriptive, with the CUQ results serving only to aid the research team in areas for improvement. Future iterations should include a larger and more diverse sample to help us obtain a better understanding of the effectiveness and of the improvements needed to the content and conversation design.

Concerning the subject matter and our approach to branched conversations based on vaccination status, our approach for fully vaccinated participants centered on information delivery. A by-product of addressing vaccine misinformation is to increase vaccination uptake, so fully vaccinated participants do not fall into this category. Chatbots for health communication deployed over social media cannot know ahead of time the characteristics of those who will use them. As such, it is important to undertake persona exercises to understand who your participants might be and include an intended outcome for each. Future studies should use an engagement strategy in which the goal is to empower fully vaccinated participants to share their experiences and information with others via social media, as well as exploring different social media strategies that might attract a greater diversity of individuals to engage with the chatbot.

Since behavior change was not in the scope of this study, integrating MI as a behavior change model needs to be explored in future iterations. MI was largely selected due to it being a well-published model in the literature about chatbots [10,54,55], but there are also those who use an eclectic approach [27] or develop and implement their own models [28-30]. Future work should include the exploration of other models of behavior change based on a larger and more diverse participant population. Additionally, a behavior change expert was not consulted in this study, and future studies would benefit from having this skill set on the development team.

We were also limited by not being able to conclude that any behavior changed; only indications of change and resistance could be detected, albeit post hoc. Previous research, such as Altay et al [56], has shown that behavior change can be detected using a chatbot; however, as the feasibility of democratizing development was the aim of this paper, future work is needed on how this might be achieved using low-cost technology solutions.

### Conclusion

This study explored the feasibility of and design and development considerations for VWise, a chatbot created to enable a greater diversity of environments to enter the chatbot space by using readily available human and technical resources. Although our findings are descriptive in nature, our pilot of VWise shows promise with regard to whether low-resource environments can enter the health communication chatbot space. The early conversational model also shows promise as many participants followed the conversation to its natural end and many extended the conversation through selection of a second concern. However, improvements to the chatbot’s personality and conversation flow are needed and further pilots with a larger sample size and diversity of vaccination status are necessary. This study represents early work of a chatbot in its virtual infancy. We hope this study will help provide those who feel chatbot access may be out of reach with a useful guide to entering this space, enabling more democratized access to chatbots for all.

## Abbreviations

CDC: Centers for Disease Control and Prevention
CUQ: Chatbot Usability Questionnaire
FAQ: frequently asked question
MI: motivational interviewing
NLP: natural language processing
SUS: System Usability Scale
WHO: World Health Organization
